# Recent Advances in ZIF Membrane: Fabrication, Separation Ability and Its Application

**DOI:** 10.3390/nano15030239

**Published:** 2025-02-04

**Authors:** Jingyuan Zhang, Jiatong Han, Xin Chen, Dan Xu, Xiaobin Wen, Yiming Zhao, Yanyan Huang, Xin Ding, Ge Chen, Donghui Xu, Xiaomin Xu, Guangyang Liu

**Affiliations:** 1 State Key Laboratory of Vegetable Biobreeding, Institute of Vegetables and Flowers, Chinese Academy of Agricultural Sciences, Key Laboratory of Vegetables Quality and Safety Control, Ministry of Agriculture and Rural Affairs of China, Beijing 100081, China; 2 College of Horticulture, Shenyang Agricultural University, Shenyang 110866, China; 3 National Center of Technology Innovation for Comprehensive Utilization of Saline-Alkali Land, Dongying 257347, China

**Keywords:** ZIF membrane, fabrication, separation, removal, water pollutants

## Abstract

With the growth of the population and the development of industry and agriculture, water resources are experiencing contamination by numerous pollutants, posing a threat to the aquatic environment and human health. Zeolitic imidazolate framework (ZIF) membranes, as a solution for water pollutant treatment, not only have the advantages of high efficiency adsorption, good selectivity, stability, and easy recyclability, but they also can be modified or derivatized through surface functionalization, compositing, or structural tuning, which can further endow the membranes with other functions, such as catalysis and degradation. In order to improve the performance of ZIF membranes, it is crucial to select suitable preparation methods to optimize the microstructure of the membranes and to improve the separation performance and stability of the membranes. This review systematically summarizes the current major preparation methods of ZIF membranes and their respective advantages and disadvantages, providing an overview of the applications of ZIF membranes in the treatment of water pollutants, such as dyes, antibiotics, and heavy metal ions. Future development prospects are also discussed, with the expectation that future research will optimize the synthesis methods to enhance the mechanical strength of the membranes and improve their selectivity, permeability, and anti-fouling properties through modifications or functionalization. This article is expected to provide theoretical support for the application of ZIF membranes in water pollution treatment.

## 1. Introduction

Human survival depends on water resources extensively, but with the development of society, the discharge of water from industrial, agricultural, and residential purposes has introduced a variety of contaminants into water, threatening human wellness and turning into the most significant environmental issue in the world [1]. Typical water pollutants, including dyes, antibiotics and heavy metal ions, transform and enrich with the food chain and water cycle [2,3]. As contaminants are endangering the aquatic environment, ecosystems and human health, it is now imperative that they be effectively removed from water.

A variety of physical techniques, chemical techniques, and biological techniques have been used for the treatment of water contaminants [4]. The application of microorganisms or plants to remove pollutants by decomposition, which can fully decompose the pollutants into CO_2_ and H_2_O, is known as biological techniques. Although they are environmentally friendly and do not contribute to secondary pollution, they are constrained by the high harm that pollutants bring to living organisms and the low bioavailability of organic pollutants, which make them unsuitable for treating hazardous wastewater [5,6]. Chemical methods are approaches that utilize oxidation or reduction to subject pollutants to chemical reactions, thereby degrading them. This category includes advanced oxidation processes, which have been a research hotspot in recent years [7]. Although the majority of chemical methods for pollutant removal are easy to operate and have high treatment efficiency, their practical applicability is still limited by drawbacks like the challenge of recycling and regenerating catalysts, low cost-effectiveness, and the potential for the introduction of new pollutants during the treatment process [8,9]. Physical treatment is a method of separating and removing pollutants by utilizing their own different physical properties, including adsorption, filtration, centrifugation and extraction [10]. It can eliminate multiple contaminants simultaneously without harsh experimental conditions. The membrane adsorption separation method, which uses membrane material to eliminate impurities from water through capture and adsorptive processes, is a widely and frequently researched technique. Particles larger than the membrane pore size of the pollutants are retained on the membrane’s surface when the wastewater containing the pollutants passes through the adsorption membrane. Smaller particles will enter the membrane and be absorbed by reactive functional groups for ion exchange, surface complexation, and other interactions, which will accomplish the goal of eliminating the pollutants from the wastewater. Adsorption membranes are exceptionally successful at eliminating a variety of newly discovered contaminants from water by combining the benefits of membrane filtration and adsorption technology [11]. However, some aspects still need to be further explored, such as membrane clogging, regeneration difficulties, high preparation cost, and possible secondary pollution to the environment. In view of these limitations, ZIF (zeolitic imidazolate framework) membranes are gaining traction as an emerging alternative.

ZIF is a porous crystalline material formed by the tetrahedral coordination of transition metal ions (such as Zn and Co) with imidazolate linkers [12], and it belongs to a category of metal–organic frameworks (MOFs) [13,14]. ZIF material possesses highly ordered porous structures, and as an emerging multifunctional material, it is being extensively explored for various cutting-edge fields [15], such as gas sensing [16], energy conversion [17], photoelectrocatalysis [18] and degradation [19]. Due to the excellent properties of ZIFs (ZIF-7, ZIF-8, ZIF-67, ZIF-71, ZIF-L, etc.), such as their abundant pore structure, large specific surface area, controllable surface texture, well-defined pore size, and diverse architectures and layouts, they can be used to adsorb contaminants such as dyes, antibiotics, and heavy metal ions from aqueous solutions [20]. ZIF has a promising future in the treatment of water pollutants. By further combining ZIF particles with a base film, a hybrid matrix film containing ZIF particles can be created. ZIF film not only encompasses the excellent adsorption properties of traditional powdered ZIF but also has many other advantages: (1) High efficiency adsorption: the ZIF membrane has a high specific surface area and a rich pore texture, which can offer an abundance of adsorption points, thus possessing high-efficiency adsorption capacity for the pollutants in water. (2) Excellent selectivity: by regulating the composition and structure of the ZIF membrane, the selective adsorption of specific pollutants can be realized to improve its purification efficiency. (3) Strong stability: the ZIF membrane has good thermal and chemical stability to maintain a stable adsorption performance under harsh environmental conditions. (4) Easily recyclable: compared with powder adsorbents, the ZIF membrane is recyclable and reusable, reducing treatment costs. Although the ZF-type membrane has a stable adsorption performance, its regeneration process is relatively complicated and requires more energy and resources. The adsorption effectiveness of ZIF membranes may deteriorate as time goes on due to contamination from other chemicals in water. Combining the capabilities of membrane adsorption and separation with organic catalytic degradation, numerous research studies in recent years have suggested that ZIF membranes can be employed as catalysts or catalyst carriers for the catalytic degradation of organic contaminants [21]. Catalytic degradation and membrane adsorption separation are organically integrated. These alterations or derivatives greatly improve the catalytic separation of pollutants in water and successfully lower membrane pollution while maintaining the benefits of ZIF materials’ high degree of porosity and high specific surface area. ZIF membranes hold great promise for efficiently detecting and eliminating organic pollutants from aquatic environments.

However, the wide application of ZIF membranes still faces many challenges, such as insufficient membrane stability and difficulty in large-area preparation. Therefore, the development of new preparation methods to overcome these challenges and enhance the performance and application range of membranes is an area of opportunity with significant research value. Currently, the major synthesis methods for ZIF-type membranes include the in situ growth method, secondary growth method, chemical vapor deposition method, interfacial polymerization method, counter diffusion method, solvothermal synthesis method, dilute solution coating method, electrochemical synthesis method, and electrospinning technology. Each of these methods has its own advantages and disadvantages, and different preparation methods can be selected according to the desired pore size, defect density and flexibility of the membrane to optimize the separation performance of ZIF membranes. In addition, with the increasing requirements on the performance of ZIF membranes, the development of new preparation methods to optimize the microstructure of the membranes and to improve the stability and separation performance of the membranes has become a hotspot of current research. Therefore, systematically summarizing and exploring the preparation methods of ZIF membranes not only helps to promote the further development of ZIF membrane preparation technology but also provides important references and lessons for researchers in related fields.

The objective of this review is to summarize the techniques for the manufacture of ZIF membranes and their advantages and disadvantages, as well as to provide assistance in the selection of preparation methods in subsequent studies. It provides ideas for applying ZIF membranes to purify water pollution by methodically summarizing the use of modified or derived ZIF membranes in water pollution treatment and anticipating the future development of their performance improvement.

## 2. Preparation of ZIF Membrane

ZIF membranes are ZIF-X hybrid matrix membranes formed by combining ZIFs as precursors with organic polymers such as polyamide (PA) [22], polyethersulfone (PES) [23], polyvinylidene fluoride (PVDF) [24], polyacrylonitrile (PAN) [25], and other organic polymers through a series of physical or chemical methods. ZIF membranes not only maintain the benefits of an expansive specific surface area, high degree of porosity, customizable pore size, and the outstanding stability of the ZIFs materials but also have the continuity and integrity that permit the selective separation of gasses and liquids. The main synthesis methods for ZIF membranes are the in situ growth method, secondary growth method, chemical vapor deposition method, interfacial polymerization method, counter diffusion method, solvothermal synthesis method, dilute solution coating method, electrochemical synthesis method, and electrospinning technology. The advantages and disadvantages of these nine ZIF membrane preparation methods are summarized in Table 1.

### 2.1. In Situ Growth Method

**Figure 1:**
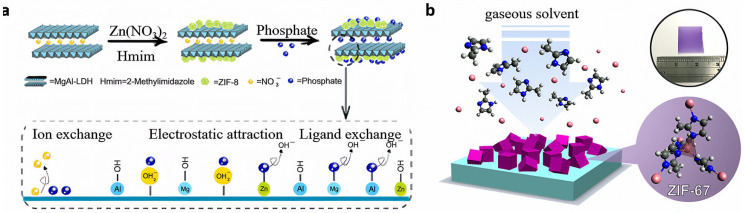
(**a**) Preparation of MgAl-LDH@ZIF-8 membrane synthesis by in situ growth method [26]; (**b**) preparation of uniform ZIF-67 thin film on a c-sapphire substrate by chemical vapor deposition [34].

ZIF materials can be grown directly on a particular substrate or membrane layer employing the in situ growth process. This method usually involves the introduction of reactants on the surface of the substrate, and the ZIF material is synthesized and grown directly on the substrate through managing the reaction circumstances. This method first requires the introduction of reactants on the surface of the substrate. These reactants typically include a metal source (such as zinc) and an organic ligand (such as 2-methylimidazole). By dissolving these reactants in an appropriate solvent, they can be evenly distributed on the surface of the substrate. Controlling the reaction conditions is key to the in situ growth method. The reaction usually needs to be carried out under hydrothermal conditions, which includes controlling the reaction temperature, time, and pressure. During the reaction process, the ZIF material will nucleate and grow on the surface of the substrate. For example, DAI et al. [26] prepared MgAl-LDH@ZIF-8 composites by growing ZIF-8 on layered double hydroxide (MgAl-LDH) via the in situ growth method for the effective process of removing phosphate from wastewater (Figure 1a). In comparison to pure ZIF-8 and MgAl-LDH, MgAl-LDH@ZIF-8 has been demonstrated to exhibit a markedly enhanced phosphorus removal efficiency reaching up to 99%, with a maximum adsorption capacity of 68.53 mgP·g^−1^. The advantage of the in situ growth method is that a structurally homogeneous ZIF film can be prepared, which does not easy detach due to it tightly bonding with the substrate. Additionally, the properties of the composite material can be optimized by adjusting the thickness and morphology of the film through the control of the reaction time and conditions [27]. The disadvantages are that the reaction conditions must be strictly controlled, the substrate may be incompletely covered, and the substrate may crack during the heating and cooling process, resulting in the detachment of the ZIF layer [28].

### 2.2. Secondary Growth Method

The secondary growth method involves forming a crystalline seed layer on the substrate and then uses the crystalline seed layer as the starting point for subsequent growth and further grows ZIF films on the crystalline seed layer through chemical reactions or physical processes [29]. The secondary growth method is frequently employed in the production of high-quality ZIF films. For example, VO et al. [30] efficiently prepared photocatalytic membranes assembled from -AOOH-PVA (BOP)-modified heterostructured ZIF-67/AgCI/Ag composites by combining the secondary growth method and photoreduction method. First, ZIF-67-inoculated BOP membranes were molded in culture and then submerged in the 2-methylimidazole ligand for secondary growth to acquire BOP/ZIF-67 membranes. Next, AgCI/Ag solution was formulated on the membrane by immersing AgCI/Ag in AgNO_3_, and then photoreduction was performed under a visible LED light to obtain BOP/ZIF/AgCI/Ag membranes. In visible light LED irradiation, the photodegradation experiment demonstrated that the BOP/ZIF/AgCI/Ag membranes effectively eliminated tetracycline (TC) and rhodamine B (RhB) dye, achieving degradation efficiencies of approximately 99% within 90 min and 95% within 140 min, with corresponding reaction rates of around 0.046 min^−1^ and 0.019 min^−1^, respectively. The benefits of this approach include the ability to create ZIF films with dense structures and high thicknesses, as well as the opportunity to modify the films’ transformations and characteristics by regulating the seed layer preparation and secondary growth conditions [31]. The disadvantages of this method are that the crystal seed layer needs to be prepared on the substrate in advance, which increases the preparation steps and necessitates many requirements for the selection and treatment of the substrate [29,32].

### 2.3. Chemical Vapor Deposition

Chemical vapor deposition is a technique employed to deposit precise, ultrathin films onto a substrate surface by chemical events in the gas phase [33]. In order to obtain ZIF membranes, metal ion and organic ligand precursor gasses are introduced into a reaction chamber, where the reaction of chemicals takes place on the substrate. Specifically, the reactants are introduced into the reaction chamber in a gaseous form and are subsequently activated within the chamber, typically through heating or plasma treatment. The activated reactants then undergo chemical reactions on the surface of the substrate, leading to the formation of a thin film. As additional reactants continuously enter the reaction chamber and react with the substrate surface, the thin film gradually grows. The formed film adheres to the substrate surface and crystallizes, ultimately resulting in a ZIF film with a specific morphology and structure. For example, Huang et al. [34] reported a steam-assisted chemical vapor deposition method for the straightforward synthesis of highly crystalline ZIF-67 membranes in the range of 95 to 125 °C (Figure 1b). The technique incorporates vertically aligned ZIF-67 as the active component in chemiresistors on microelectronic devices, and the ZIF-67-based chemiresistors can detect gas molecules, which are able to adsorb gas at an ambient temperature into the ZIF- 67 cages. High purity, high densification, and good crystallinity are the benefits of chemical vapor deposition films, which may even be made on substrates with complex shapes [35]. The disadvantage is the high reaction temperature [33], which may damage the substrate material. The equipment used is complex, resulting in high molding costs. In addition to this, the process of creating films requires the precise control of conditions such as temperature and gas flow.

### 2.4. Interface Polymerization Method

Interfacial polymerization is the conventional approach for the laboratory preparation of reverse osmosis and nanofiltration membranes. The interfacial polymerization method is fundamentally based on the reaction between two different monomers, with one being soluble in the aqueous solvent and the other in the organic solvent. Upon contact between the aqueous and organic phases on a porous membrane, a polymer film develops on the substrate’s surface. As the polymer film grows, it prevents further interaction between the two reactants, leading to the creation of an ultra-thin, selective barrier [36]. Based on the traditional interfacial synthesis method, LI et al. [36,37] introduced a novel approach for fabricating continuous MOF membranes through interfacial MOF synthesis by using PES-based ultrafiltration membranes as carriers and selecting ZIF-8 as a representative example. PES porous membranes, as a kind of flexible polymer, have good interaction with organic ligands of MOFs, which can be used as a basis for the synthesis of other types of continuous ZIF membranes. LIN et al. [38] deposited a homogeneous and selective ZIF-8 layer on the surface of HPAN by the interfacial polymerization method so that the hybrid membrane combined the beneficial properties of ZIF-8 with those of HPAN. The membrane can remove more than 90% of the organic matter in humic acid (HA) solution, and the membrane surface coated with the ZIF-8 layer is uniformly distributed. This makes it possible to grow the ZIF-8 layer directly onto the polymeric support at room temperature, which can form a dense ZIF layer at the interface and solve the problems of particle agglomeration and uneven film thickness. The downside of this approach is the necessity for stringent control over reaction conditions to avoid the formation of defects at the interface.

### 2.5. Counter Diffusion Method

The counter diffusion method mainly utilizes the diffusion of reactant molecules from different phases through the base film or support layer to the other side and reacts at the interface to form a ZIF membrane. This method usually involves placing solutions containing metal ions (e.g., zinc ions) and organic ligands (e.g., imidazole ligands) on each side of the base film, where they meet and undergo a coordination reaction by diffusion, resulting in the growth of a homogeneous ZIF film layer [39,40]. HUANG et al. [41] successfully prepared hydrophobic ZIF-71 hollow fiber membranes via the reverse diffusion method, and the prepared membranes had very high integrity. Due to its excellent affinity for organic matter, the ZIF-71 hybrid membrane demonstrated superior ethanol recovery capabilities. The advantages of the counter diffusion method are that it can ensure that the ZIF membrane grows uniformly over the substrate membrane’s exterior, which can reduce the non-uniformity of the thickness of the membrane; by controlling the reaction conditions and the diffusion rate, the ZIF membrane can be prepared with dense structure and no defects, which contributes to improving the separation performance of the membrane [42]; and by varying the type and concentration of the reactants as well as the reaction conditions, it is possible to control the pore size, the void ratio and the specialized surface area of the ZIF membrane, as well as other parameters, to satisfy the needs of different requirements [43]. The disadvantages of the counter diffusion method are that it requires the precise control of a variety of reaction parameters, such as the concentration of reactants, diffusion rate, reaction temperature and time, which makes the operation relatively complicated; the whole preparation process may be relatively time-consuming because of the need to rely on the diffusion effect to make the reactants meet and react at the interface.

### 2.6. Solvothermal Synthesis Method

The solvothermal synthesis method involves using an organic solvent or water as the reaction medium within a sealed reaction system to facilitate reactions at elevated temperatures and pressures. By controlling the reaction parameters, including temperature, and duration, the target product can be synthesized. In the preparation of ZIF membranes, solvothermal synthesis is commonly used to grow ZIF crystals on a substrate, forming a thin film [44,45]. For example, Sahu et al. [46] synthesized Co-ZIF membranes through the solvothermal synthesis method, which involved heating the material in an inert atmosphere followed by an acid-leaching step to selectively remove Co. They utilized the conductivity and structural selectivity of this C-based structural material for the electrocatalytic oxidative coupling of isobutylene and styrene. The advantages are the ability to control the growth rate and morphology of the crystals, the preparation of high-quality ZIF membranes, and the simplicity of the operation, which makes it easy to prepare on a large scale [47]. The disadvantages are that it requires high temperature and pressure, high energy consumption, and possible by-products.

### 2.7. Dilute Solution Coating Method

The dilute solution growth method is a technique that utilizes an ultra-low concentration of precursor solution to coat a ZIF film on a substrate. By controlling the concentration of the solution and the reaction conditions, a loosely structured macroporous coating can be formed on the substrate. For example, LI et al. [48] used the dilute solution coating method to form a porous and water-repellant ZIF-71/PVDF layer on the exterior of PVDF hollow fiber-supported membranes, which further improved the hydrophobicity of the PVDF membranes and endowed the coatings with particular adsorption capabilities, thus elevating the membrane permeability. Simple operation, cheap cost, and the ability to work in a room-temperature solution are among the benefits of this method. The membrane’s thickness can be modified by adjusting the concentration of the solution and the number of coating cycles, making it appropriate for large-scale manufacturing. The drawbacks include the difficulty of controlling solution concentration and reaction conditions, as well as the possibility that solvent evaporation and the reaction time will impact the film’s thickness and homogeneity.

### 2.8. Electrochemical Synthesis Method

**Figure 2:**
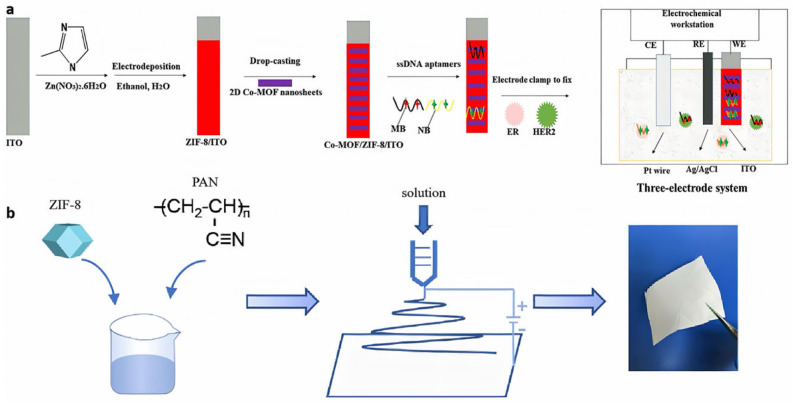
(**a**) Preparation of ZIF-8 nanofilms by electrochemical synthesis [50]; (**b**) preparation of PAN/ZIF-8 nanofibrous membranes by electrostatic spinning technique [25].

Electrochemical synthesis is a method of producing a ZIF film by deposition on an electrode surface using electrochemical means [49]. This method usually involves the directional migration and reaction of metal ions and organic linkers under the action of an electric field, leading to the formation of a ZIF film on the electrode surface with specific structure and properties. For example, Zhang et al. [50] developed an ultrasensitive electrochemical aptamer sensor based on Co-MOF/ZIF-8 nanofilms by cathodic synthesis via electrochemical methods (Figure 2a), where the amount of aptamer chains could be adsorbed on indium tin oxide (ITO) slices through the electrical conductivity of Zn-MOF thin films, the specific surface area, and the ease of assembling the two-dimensional Co-MOF nanosheets. The aptamers were labeled by the electrostatic binding of neutral red (NB) and MB, respectively. The advantages of electrochemical synthesis are that the preparation method is simple and the conditions are mild; under the action of the electric field, the crystal composite structure can be uniformly formed on the surface of the base film, resulting in the uniform thickness of the ZIF separation layer and fewer defects [51]; under the stabilized electric field strength, the growth of the thickness of the ZIF separation layer has good self-limitation, which is conducive to the control of the thickness of the membrane layer; and it usually has lower energy consumption and produces fewer wastes compared with other synthesis methods. The disadvantages are that in order to ensure the quality and performance of ZIF membranes, specific electrochemical equipment and precise control conditions are required, and the cost of equipment and maintenance may be high and the process control complicated.

### 2.9. Electrospinning Technology

Electrospinning is a technique that uses electrostatic field forces to stretch polymer solutions or melts into nanofibers. The technique involves stretching polymer solutions or melts into ultrafine fibers under the action of an electric field, with the fibers then collected on a collector to form a membrane. Electrospinning technology is usually not directly used in the producing of ZIF membranes but can be used to prepare composite fiber membranes containing ZIF structures [52]. Wang et al. [25] prepared a PAN/ZIF-8 nanofiber membrane by the electrostatic spinning technique (Figure 2b). In this process, ZIF-8 nanoparticles were integrated into the nanofibers, creating a bead-like configuration that allowed ZIF-8 to be accessible on the nanofiber surface, resulting in the effective adsorption of methyl blue (MB). The advantage of the electrospinning technique is that the prepared membranes have a larger specific surface area, higher porosity and larger pore size [53]. In addition, the diameter and form of the fibers can be meticulously regulated by fine-tuning the spinning parameters, and it is possible to prepare membranes containing a variety of materials [54]. The disadvantages are that there may be problems such as uneven fiber thickness and inter-fiber adhesion during the spinning process [55]; the preparation efficiency is relatively low, and the preparation process may produce environmental pollution.

**Table 1:** Comparison of advantages and disadvantages of 9 preparation methods for ZIF membranes.

Methodologies	Advantages	Disadvantages
In situ growth method	ZIF films are tightly bonded to substrate and have a homogeneous structure; thickness and morphology can be controlled [27]	Possible incomplete coverage of substrate; possible cracking of substrate during heating and cooling [28]
Secondary growth method	Preparation of ZIF membranes with large thickness and dense structure; adjustable morphology and properties of the membranes [31]	Requires pre-preparation of crystal seed layer on substrate, which increases preparation steps; high requirements for substrate selection and treatment [29,32]
Chemical vapor deposition	High purity, good densification and good crystallinity of prepared films; films on complex-shaped substrates can be prepared [35]	Higher reaction temperatures may damage substrate material; complex equipment and high molding costs; precise control of temperature and gas flow required
Interface polymerization method	Can be prepared at room temperature; ZIF layer is dense and uniformly distributed [38]	Reaction conditions need to be tightly controlled to avoid formation of defects at interface
Counter diffusion method	Homogeneity and densification of prepared ZIF membranes; performance parameters of ZIF membranes can be tuned [43]	Requires precise control of multiple reaction parameters and is relatively complex to operate; preparation can be relatively time-consuming
Solvothermal synthesis method	Controllable crystal growth rate and morphology for high-quality ZIF films; simple operation and easy for large-scale film production [46]	Demands stringent temperatures and pressure settings, leading to significant energy usage; may produce by-products
Dilute solution coating method	Simple operation and low cost; can be carried out in a room temperature solution; thickness of film can be regulated by managing concentration of solution and number of coats, which is suitable for mass production of ZIF films [48]	Higher requirements for solution concentration and reaction condition control; film thickness and uniformity may be affected by solvent evaporation and reaction time
Electrochemical synthesis method	Simple and mild preparation method [51]; uniform ZIF film thickness and fewer defects; good control of film thickness; usually lower energy consumption and less waste generation [50]	Requires specific electrochemical equipment and precise control conditions; equipment and maintenance costs can be high; process control is complex
Electrospinning technology	Has larger specific surface area, enhanced porosity and more substantial pore dimensions [53]; fiber diameter and morphology; fiber membranes containing various materials can be prepared [54]	There may be uneven fiber thickness, inter-fiber adhesion and other problems [55]; preparation efficiency is relatively low; the preparation process may produce environmental pollution

## 3. Application of ZIF Membranes in the Removal of Water Pollutants

### 3.1. Adsorption

ZIF materials have many advantages over other types of MOF materials for the adsorption of pollutants: (1) ZIF materials have excellent thermal and chemical stability and are able to maintain structural stability in extreme environments. This makes them more durable and reliable in dealing with a wide range of pollutants. (2) They have high porosity and a large specific surface area, which increases their chances of coming into contact with contaminants, thereby improving their adsorption efficiency. (3) The surfaces of ZIF materials can be tuned through chemical modification to enhance the adsorption of specific pollutants. This tunability of their surface functionality allows ZIF materials to be adapted to different pollution treatment needs. (4) ZIF materials can be prepared as multifunctional composites by compositing them with other materials and combining the advantages of multiple materials to improve the overall performance. ZIF materials show great potential and advantages in the adsorption of pollutants due to their unique physicochemical properties. The most basic application of the ZIF membrane in the removal of water pollutants is to utilize the membrane’s retention and adsorption properties. When the wastewater containing pollutants passes through the adsorption membrane, the pollutants with particle sizes beyond the pore dimensions of the membrane are retained on the surface of the membrane; the pollutants with a smaller particle size enter into the membrane and interact with the reactive functional groups inside the membrane in various ways, such as via ion exchange or surface complexation, so as to achieve the purpose of removing the pollutants in the wastewater [11].

#### 3.1.1. Adsorption of Dyes

Dyes are widely utilized across industries such as dyeing, printing, textiles, leather processing, and ink production. The most significant dye kinds utilized in the textile sector are azo, anthraquinone, and phthalocyanine dyes [56]. Due to incorrect or careless handling during their manufacture and use, dyes may end up in water bodies, where they pollute soil by using water as a medium. Most of the organic dyes and their metabolites are biotoxic and carcinogenic, which can be detrimental to aquatic life, microorganisms and human health [57]. Membrane adsorption, as a physical technique for the removal of organic dyes from aqueous environment, has drawn significant research interest because of its advantages of being renewable and easy to operate.

ZIF membranes mainly rely on non-covalent forces to adsorb dye molecules, such as *π-π* stacking interactions and electrostatic interactions. However, the hydrogen bonding and ligand bonding effects in covalent interactions may have a small contribution to the capture of dye molecules.

**Figure 3:**
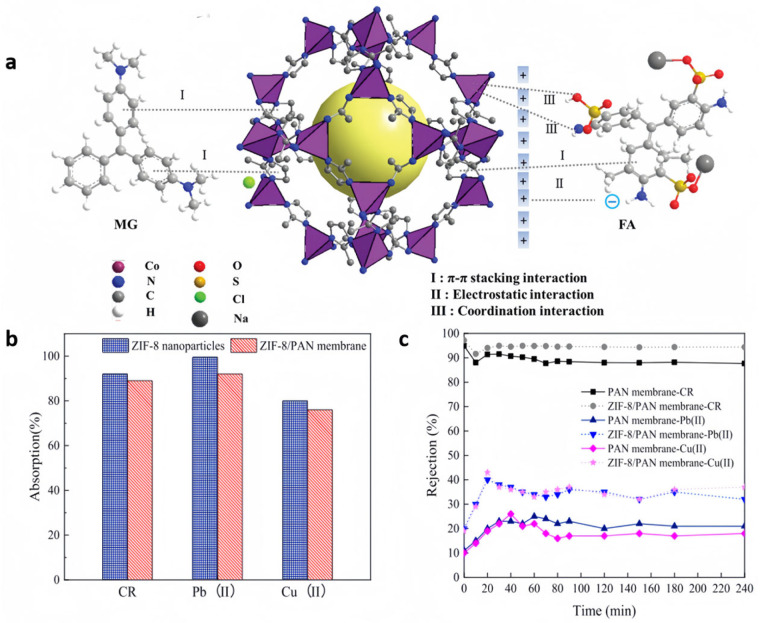
(**a**) Suggested pathways for MG and FA adsorption onto ZIF-67/PVDF composite membranes [24]; (**b**) static adsorption of CR, Pb(II) and Cu(II) by ZIF-8/PAN composite membrane and ZIF-8 nanoparticles, with test parameters set at 0.1 g/L for nanoparticles, 0.2 g/L for the membrane, and 25 mg/L for starting contaminants [58]; (**c**) exclusion capability of CR, Pb(II) and Cu(II) by ZIF-8/PAN membrane and PAN membrane, tested at initial pollutant concentration of 25 mg/L and transmembrane pressure of 0.01 MPa [58].

Liu et al. [24] combined the surface activity of ZIF-67 and the excellent chemical, mechanical and thermal characteristics of PVDF membranes to prepare ZIF-67/PVDF hybrid membranes for the adsorption of two triarylmethane dyes, malachite green (MG) and acidic magenta (FA). The ZIF-67/PVDF hybrid membranes showed the good selective adsorption of MG and FA, with adsorption capacities of 6974.48 μg·cm^−2^ and 4636.80 μg·cm^−2^. The robust adsorptive capacity of the ZIF-67/PVDF membranes for FA and MG can be attributed primarily to the intense *π-π* stacking interactions between the aromatic rings of FA and MG and the imidazole ring of ZIF-67 (Figure 3a). Moreover, hydrogen bonding could also play a role in enhancing the adsorption efficiency, but not much. In particular, ligand bonding effects and electrostatic interactions between -NH_2_ on FA and Co^2+^ in ZIF-67 may also lead to the adsorption of FA.

Li et al. [58] employed the electrospinning technique to prepare ZIF-8/PAN composite membranes for the removal of Congo red (CR), Pb(II) and Cu(II) from industrial wastewater, which was favorable for the removal of CR in an acidic environment (pH = 2). Among them, the ZIF-8 nanoparticles in this membrane had an adsorption capacity of up to 700 mg CR·g^−1^, with an 89% adsorption rate, for CR via van der Waals forces, electrostatic interactions, hydrogen bonding, and *π-π* interactions (Figure 3b,c).

Sun et al. [59] employed an in situ growth method to add ZIF-L material to electrostatically spun polyacrylonitrile nanofibers (NFs), and the resulting ZIF-L/NF nanocomposite membranes efficiently adsorbed the toxic dye MG, with an adsorption ability of 5103 mg·g^−1^. This high adsorptive power is due to the *π-π* stacking interactions between ZIF-L and the dye molecules. The ZIF-L/NF membranes were found to be stable in the range of pH 3–7 in terms of their adsorption capacity and showed excellent recoverability in a five-cycle test of adsorption–desorption. This result confirms the stability of ZIF-L/NF membranes for the elimination of dyes from water.

#### 3.1.2. Adsorption of Antibiotics

Antibiotics have been widely used since their discovery to treat human and animal diseases due to their inhibitory effect on bacterial growth and reproduction [60]. To date, several types of antibiotic drugs are available, such as tetracyclines, sulfonamides, beta-lactams, quinolones, and penicillin [61]. However, as antibiotics are non-biodegradable and toxic, residues in water bodies may inhibit or affect microorganisms and aquatic organisms in the water, causing serious environmental and aquatic ecosystem problems [62]. Similar to dye molecules, the adsorption forces of antibiotics by ZIF membranes include ligand bonding, hydrogen bonding, *π-π* bonding, and electrostatic interactions.

Wu et al. [63] introduced an efficient method for fabricating oriented ZIF-L adsorption membranes on commercial polymer substrates through a straightforward room-temperature immersion technique. The self-polymerization property of polydopamine (PDA) was first utilized to create an interface on PVDF, and then the ZIF-L layer was grown in situ to obtain ZIF-L composite membranes. The membrane-grown adsorbent exhibited a greater adsorptive capacity compared to the crystals prepared in bulk solution, with a static adsorption of 196 mg·g^−1^ of tetracycline (TC) at 30 °C.

In Li et al.’s study [64], polylactic acid (PLA) electrostatically spun fibers and ZIF-8 were used to prepare PLA/ZIF-8 composite membranes. The optimal PLA/ZIF-8 membrane was selected for antibiotic adsorption tests on tetracycline hydrochloride and oxytetracycline (OTC) at different temperatures. The results demonstrated that an elevated temperature and higher initial concentrations in the liquid phase may diminish the membranes’ adsorptive performance and target molecules. The maximum adsorption of tetracycline hydrochloride (TCH, 50 mg/L) and OTC (40 mg/L) at 60 °C was 90.92 and 69.64 mg·g^−1^, respectively.

Wang et al. [65] designed and prepared a ZIF-8-decorated regenerated cellulose nanofiber membrane (ZIF-8@RC NFM) by integrating electrostatic spinning with in situ surface modification techniques. Due to its good surface wettability, improved tensile properties, interlinked porosity and substantial specific surface area, the obtained ZIF-8@RC NFMs showed a significant adsorption ability of 105 mg·g^−1^ for TCH within 3 h, which provides a basis for the advancement of adsorbents used in wastewater treatment. The membrane’s adsorptive action hinges on the coordination bonds formed between the metal active sites on ZIF-8 particles and the highly electronegative atoms within the TCH molecules, facilitating the adsorption of TCH onto the ZIF-8@RC-3 NFMs’ surface. In addition, hydrogen bonds and *π-π* bonds along with electrostatic interactions between ZIF-8 particles and TCH molecules, influence the adsorption of TCH molecules onto the ZIF-8@RC-3 NFMs’ surface.

#### 3.1.3. Adsorption of Heavy Metals

Prolonged release of untreated or poorly treated wastewater from industrial and agricultural sources is leading to severe contamination of water bodies by heavy metals [66]. Heavy metals, including copper (Cu), mercury (Hg), arsenic (As), lead (Pb), cadmium (Cd), and chromium (Cr), are known to persist in the environment and bioaccumulate in living organisms’’ tissues, and can be magnified through the food chain, threatening the health of animals and humans [67]. ZIF membranes purify wastewater containing heavy metals through physical and chemical adsorption.

**Figure 4:**
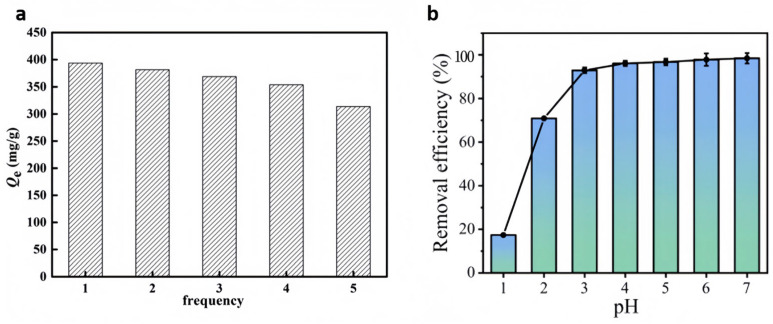
(**a**) Regeneration of f-ZIF-8@GO [68]; (**b**) effect of pH on Hg(II) adsorption by the ZIF-71/MWCNT membranes [69].

In the case of the physical mechanism, the porous nature of the ZIF membrane acts as a barrier to larger heavy metal ions, physically retaining them while permitting the smaller water molecules to get through. The negatively charged ZIF’s surface can adsorb cationic metal ions via electrostatic interaction. In addition, the extensive surface area of ZIF nanoparticles provides many reactive sites for capturing heavy metal ions. For example, Wei et al. [68] developed f-ZIF-8@GO composite membranes using ZIF-8 and graphene oxide (GO), which were functionalized with 3-aminopropyl-triethoxysilane (APTES) via amine modification, and applied them to the adsorption of Cu^2+^ in water. The equilibrium adsorption ability of the membrane for Cu^2+^ was as high as 1872.24 mg·g^−1^, which showed a strong affinity for copper ions and the ability to be recycled as an adsorbent, with sustained strong adsorption capability over multiple uses. The adsorption thermodynamics of this adsorption process was studied at three distinct temperature levels with Gibbs free energy (ΔG) values ranging from −20 to 0 kJ·mol^−1^, indicating that the adsorption process is predominantly physical in nature. The prepared material has high selectivity for Cu^2+^, can be reused many times and maintains intense adsorptive efficiency (Figure 4a).

On the other hand, through a chemical mechanism, ZIFs can engage in ion exchange reactions with heavy metal ions present in water through their functional groups, thus effectively removing them from water. Furthermore, the functional components present on the surface of ZIF particles can also undergo complexation reactions with metal ions, and this complexation can form stable complexes that prevent heavy metal ions from returning to aqueous solution. For example, Shao et al. [69] combined ZIF-71 with multi-walled carbon nanotubes (MWCNTs) to prepare defect-free ZIF-71/MWCNT membranes with improved mechanical properties, exceptional electrical conductivity, and increased adsorption capacity. The ZIF-71/MWCNTs membranes efficiently adsorbed Hg(II) in the pH range of 3–7 (Figure 4b), with an adsorption ability of 579.6 mg·g^−1^ and a dynamic adsorption performance of over 400 mL, and had good regeneration properties. The characteristic amount (E) calculated from the D-R isothermal curve fitting results was 10.66 kJ·mol^−1^, pointing that the adsorption involved the chemisorption process. In addition, it enables the swift and dependable electrochemical sensing of Hg(II), featuring a broad linear response from 0.1 to 200 μg·L^−1^ and a minimal detection threshold of 0.047 μg·L^−1^.

Gozali et al. [70] developed a polysulfone (PSU)-based ZIF-8 nanocomposite membrane for decontaminating metal ions in wastewater. Studies related to heavy metal removal showed that the 14 nm PSU/ZIF-8 membrane removed 96.25%, 90.56%, 78.81%, and 67.10% of Pb, Cu, Ni, and Cr, respectively, at a pressure of 7 bar, which demonstrated satisfactory performance in practical heavy metal removal situations.

### 3.2. Catalytic Degradation

ZIF membranes function as catalysts or catalyst carriers for the catalytic or photocatalytic breakdown of organic pollutants. The strong thermal and chemical reliability of ZIF materials, along with their excellent ability in degrading organic compounds upon visible light exposure, position them as a promising candidate for developing self-cleaning membranes (Table 2). By introducing specific metal ions or organic ligands, the properties and structure of ZIF can be modulated to provide excellent catalytic activity; the metal ions and organic ligands in ZIF can form active sites to adsorb and activate organic pollutant molecules. Under light or heating conditions, these active sites can promote the oxidation or reduction reaction of organic pollutants, thus degrading them into harmless substances. The mechanism of pollutant degradation mainly involves electron migration and the action of functional groups.

The electron migration process is due to the semiconductor nature of ZIF, which is capable of generating photogenerated carriers (electrons and holes) under light [71]. These photogenerated carriers can transfer to the surface of the material and participate in the decomposition reaction of pollutants [72]. For example, in the photocatalytic breakdown of organic contaminants, photogenerated electrons and holes produced from light can collaborate with O_2_ and H_2_O, respectively, to generate reactive oxygen species (ROS) like hydroxyl radicals (·OH) and superoxide anion radicals (O2–), which are capable of oxidizing and decomposing organic pollutants. Organic ligands in ZIF materials usually contain specific functional groups, and these functional groups can enhance the light-driven catalytic performance. For example, isocyanate (–N=C=O) groups are photoresponsive within the span of 350–450 nm, which enhances the absorption of visible light by the material, while sulfhydryl-complexed Cu (–SCu) groups can widen the visible light absorption range of MOFs and promote the generation and migration of photogenerated carriers, which can improve the photocatalytic efficiency [73].

Functional groups can also act as active sites to react directly with pollutant molecules. The surface active sites provided by unsaturated metal ions and organic ligands in ZIF materials can form coordination bonds with pollutant molecules, immobilize the pollutants on the exterior of the materials by chemisorption, and subsequently undergo degradation reactions under the action of photogenerated carriers. ZIF, through electron migration and the action of functional groups, can effectively enable the degradation of pollutants, which can mitigate the contamination of the ZIF membrane. This concept holds great potential for achieving the efficient removal of pollutants from water bodies.

**Figure 5:**
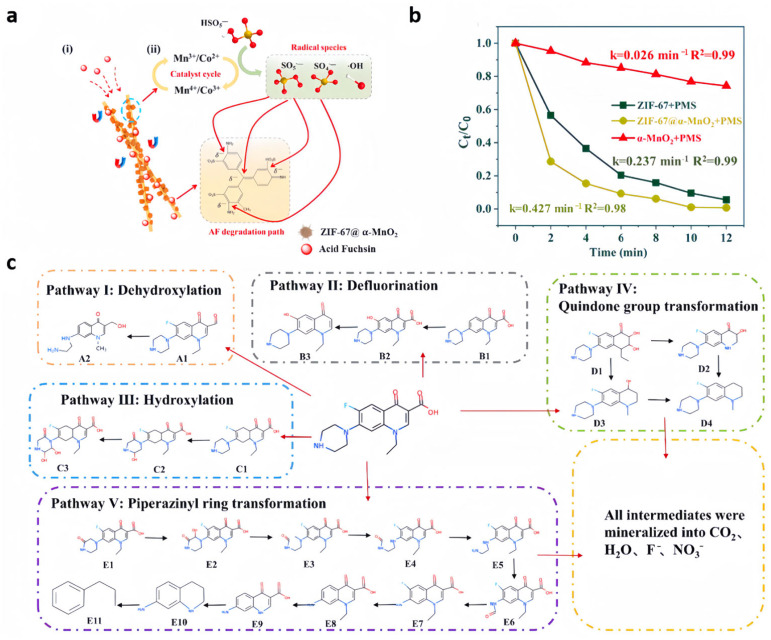
(**a**) Principle of AF elimination by NFM/PMS systems [74]; (**b**) The catalytic capability of ZIF-67, α-MnO_2_ and ZIF-67@α-MnO_2_ for AF [74]; (**c**) suggested partial degradation pathway for NOR within ZnCo-ZIF/PAN/PMS oxidation system [75].

Wang et al. [74] equipped a ZIF-67@α-MnO_2_ nanofibrous membrane (NFM) via the in situ growth method and electrostatic spinning method. The wattle-spherical ZIF-67@α-MnO_2_ nanoparticles have quite strong adsorption properties, which can promote the delivery of reactants to the catalyst surface and consequently lead to the efficient breakdown of organic contaminants (Figure 5a). Moreover, the electrostatic spinning technique can firmly fix the nanocatalysts within the nanofibers, preventing their self-aggregation. This feature simplifies the retrieval of the catalyst particles and helps prevent further water contamination. Benefiting from the abundant active sites provided by ZIF-67@α-MnO_2_ and the enhanced electron transfer capability of the nanoparticles, the optimal NFM nanofiber membranes possessed a considerable adsorption capacity of 2495 mg·g^−1^ and an exceptional catalytic efficacy, degrading 99% AF in 20 min, and they also showed a remarkable response to AF (Figure 5b). In addition, the obtained membranes showed commendable multifunctional catalytic performance against TC (95.3%), norfloxacin (NOR, 98.4%) and doxycycline hydrochloride (89.2%).

Li et al. [75] successfully immobilized ZnCo-ZIF on electrostatically spun PAN nanofibers by the in situ growth method with water as a solvent to form dense vine-like composite nanofiber membranes for the activation of peroxynitrite (PMS) to degrade NOR (Figure 5c). The ZnCo-ZIF/PAN fibrous membranes have a large specific surface area, which contributes to the homogeneous and effective exposure of the metal active sites. The findings indicated that ZnCo-ZIF/PAN was particularly effective at activating PMS, resulting in a 93% degradation of NOR degradation in just 120 min. The effectiveness of NOR removal escalated with the elevation of the Co doping level. The catalyst exhibited considerable activity across the pH range from 3 to 7. Significantly, the flexible ZnCo-ZIF/PAN composite nanofiber membrane can be fully retrieved from the treated wastewater, effectively addressing the recovery and recycling of nanopowder. This study offers novel insights into the development of bimetallic ZIF composite fibers with superior catalytic characteristics, presenting innovative concepts for their implementation in real-world manufacturing processes.

Photocatalysis is deemed a promising approach for environmental purification. The strong thermal and chemical stability of ZIF-8, as well as its excellent ability in breaking down organic molecules when exposed to visible light, makes it highly promising for developing self-cleaning membranes. Metal ions and organic ligands in ZIF membranes can form active sites that adsorb and activate organic pollutant molecules. Under light or heat conditions, these active sites can promote the oxidation or reduction of organic pollutants, thereby degrading them into harmless substances.

**Figure 6:**
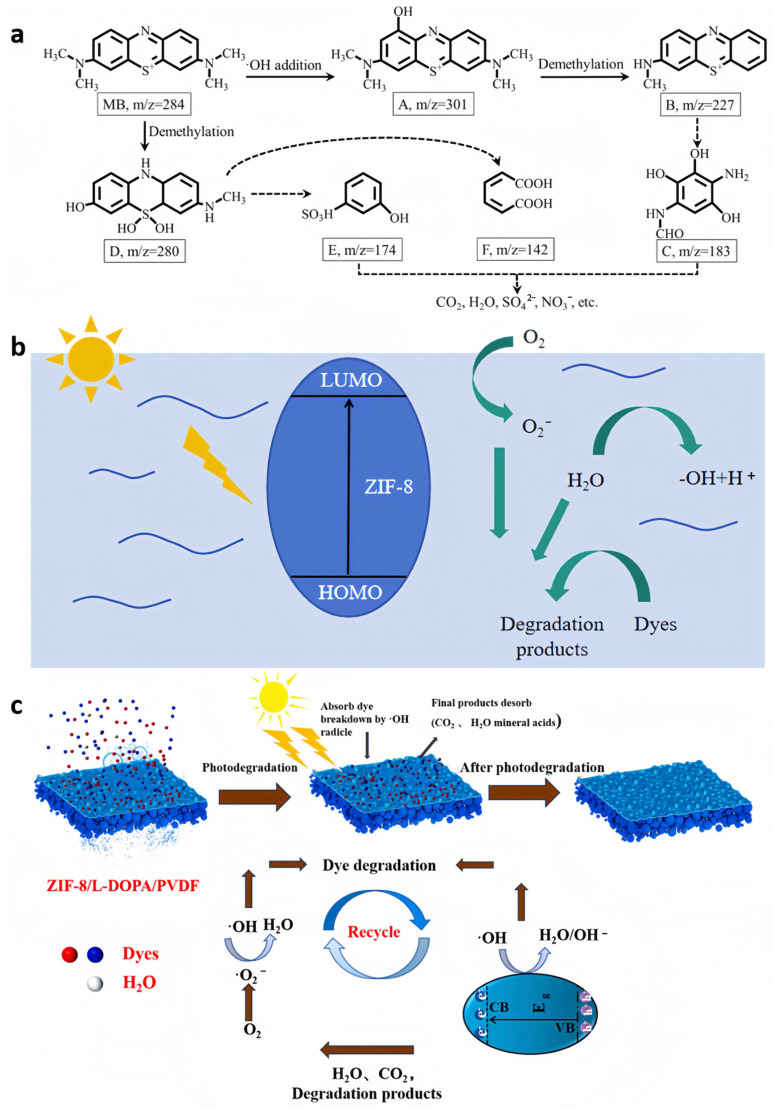
(**a**) Possible photocatalytic degradation pathway of MB [76]; (**b**) the photocatalytic degradation mechanism of dyes by PTFE@ZIF-8 membrane [77]; (**c**) photocatalytic process of the ZIF-8/L-DOPA hybrid matrix membrane [78].

He et al. [76] constructed a ZIF-8 photocatalytic membrane and its derivative ZnS photocatalytic membranes by a simple in situ process on a cellulose base, using filter paper as a representative substrate (Figure 6a). The nanomaterials could be securely affixed to the filter paper through chemical interactions. Under visible light irradiation, the designed ZIF-8 membranes and ZnS membranes achieved superior photocatalytic performance in flowing systems for the degradation of methylene blue (MB) with 97% efficiency in 80 min (k= 0.042 ± 0.002 min^−1^) and the reduction of Cr (VI) reaching 100% in 60 min (k = 0.116 ± 0.007 min^−1^). In addition, the photocatalyst membranes showed strong photostability and could be reused effectively over five consecutive cycles. Given the superior MB adsorptive performance and simple regeneration of the ZIF-8 photocatalyst membranes, this combined adsorption–degradation approach holds great potential for general application, demonstrating the considerable advantages of monolithic membranes in the photocatalytic purification of water.

Li et al. [77] successfully developed PTFE@ZIF-8 fibrous membranes for the effective photocatalytic breakdown of organic pollutants and oil–water separation in harsh environments. They electrospun polytetrafluoroethylene (PTFE) nanofibers and then grew highly porous ZIF-8 on them in situ (Figure 6b). The PTFE@ZIF-8 membrane, known for its high specific surface area similar to that of a lotus leaf, demonstrated an effective photocatalytic degradation of organic contaminants, including methylene blue (MBT), rhodamine B and TC, even in severe conditions like strong acids or alkalis, with an efficacy of 99.99% for removal. This multifunctional membrane with excellent tolerance to harsh environments provides an enlightening approach for effective wastewater management in challenging environments and holds significant potential for real-world use.

He et al. [78] constructed ZIF-8/L-DOPA/PVDF hybrid matrix membranes with remarkable self-cleaning features using DOPA as a modification by the in situ growth method (Figure 6c). The top-performing ZIF-8/L-DOPA/PVDF membrane showed an enhanced water permeability of 159.63 L·m^−2^h^−1^bar^−1^ and the excellent retention of dyes and antibiotics (CR 97.3%, MBT 98.32%, MB 98.62%, TC 95.3% and ciprofloxacin 93.34%). In addition, the distinctive attributes of ZIF-8 helped to enhanced the degradation of dyes and antibiotics by the composite membrane, thereby conferring self-cleaning capabilities. Irradiated with visible light, the composite membrane could readily eliminate adhering dirt through photocatalysis, with its flux nearly returning to initial levels after three cycles. The cyclic photodegradation of the hybrid matrix membrane can simultaneously restore the separation performance and provide excellent stability and long-term nanofiltration stability for the modified membrane.

**Table 2:** ZIFs for catalytic degradation of pollutants.

ZIF type	Metal Center	Ligand	Framework	Target Pollutants
ZIF-67	Co^2+^	2-Methylimidazole	rhombic dodecahedron [79]	AF, TC, NOR, doxycycline hydrochloride [74]
ZnCo-ZIF	Zn^2+^, Co^2+^	2-Methylimidazole	rhombic dodecahedron	NOR [75]
ZIF-8	Zn^2+^	2-Methylimidazole	three-dimensional dodecahedron	MB [76], MBT, rhodamine B, TC [77], CR, ciprofloxacin [78]

## 4. Conclusions

This review provides a comprehensive overview of the latest advancements in the fabrication of ZIF membranes, which have garnered significant interest due to their distinctive architectures and superior attributes. The synthesis methods are diverse, including but not limited to in situ growth, secondary growth, interfacial polymerization, chemical vapor deposition, reverse diffusion, solvothermal synthesis, dilute solution coating, electrochemical synthesis, and electrostatic spinning technology. The combined use of these methods provides a wealth of options for the preparation of ZIF membranes. While exploring various synthesis methods in depth, this review also systematically summarizes the advantages and disadvantages of ZIF membranes. The advantages are mainly reflected in the high porosity, good chemical and thermal resilience, and adjustable pore dimensions, which make ZIF membranes suitable for a broad spectrum of potential uses in separation and catalytic processes. Nevertheless, there are some challenges pertaining to ZIF membranes, such as their relatively low mechanical strength and demanding preparation process, which need to be solved in the subsequent research. In addition, the applications of ZIF membranes are summarized. In the treatment of water pollutants, ZIF membranes show unique advantages. Their high porosity and adjustable pore size enable ZIF membranes to effectively retain and remove contaminants in water, such as organic dyes, antibiotics, and heavy metal ions. In addition, ZIF membranes are chemically stable, thermally stable and reusable, enabling them to maintain a stable performance under harsh water conditions.

In order to enhance the efficiency and applicability of ZIF membranes, future research should continue to address the challenges and shortcomings of ZIF membranes. ZIF membranes can be modified or derivatized by introducing new functional groups, adjusting the pore size, or changing the surface properties of the membranes to give them a more excellent performance. These modified-derivative membrane technologies show a broad application prospect in fields such as water pollutant treatment. For example, the mechanical strength of membranes can be improved by optimizing the synthesis method, the selectivity and permeability of membranes can be augmented by incorporating new functional groups, and the anti-pollution performance of membranes can be improved by surface modification. These performance improvement directions will provide strong support for ZIF membranes in a wider range of applications. By choosing appropriate synthesis methods and modification derivation techniques, ZIF materials with excellent performance can be prepared and applied for water contaminate removal to realize the effective removal of pollutants in a green and sustainable way. This approach will provide new ideas and methods to solve the current serious water pollution problems.
